# Correlation between diabetes mellitus and patterns of alveolar bone defects in patients seeking dental implants: A cross-sectional CBCT study

**DOI:** 10.1097/MD.0000000000046245

**Published:** 2025-12-19

**Authors:** Manea Musa M. Alahmari

**Affiliations:** a Department of Periodontics and Community Dental Science, College of Dentistry, King Khalid University, Abha, Saudi Arabia.

**Keywords:** alveolar bone defect, arch, diabetes mellitus, gender, Siebert’s classification

## Abstract

There are no previous studies that recognize a correlation between diabetes mellitus and ridge defect according to Siebert’s classification. Therefore, the aim of this cone-beam computed tomography (CBCT) study was to assess correlation between diabetic patients and pattern of bone defect according to Seibert classification among patients who attended to replace their missing teeth by dental implants. The present CBCT study was conducted in private dental clinics in the Aseer region, Saudi Arabia. All the participants complained of missing teeth and came to replace them with dental implant. The data were collected from patients’ files at the period between March 2022 and May 2024. The selected files with their CBCT in the study were arranged and classified according to alveolar bone status based on Siebert’s classification of bone defects and medical condition of patients in relation to diabetic mellitus. Chi-square test was used to check the association between categorical variables. A *P*-value < .05 was considered statistically significant. For all 1500 CBCT patients according to Siebert’s classification of bone defects diabetic patients which represent 375 of participants, had highest prevalence of class II alveolar bone defect with (51.2%) followed by class III (42.4%) and significant differences were recorded in variables as gender, area of bone defect, and diabetic status of the patient with *P*-values .004, .000, and .000, respectively. In our study population, according to Siebert’s classification of bone defects, CBCT of the contributors shows that class II was the highest prevalence in all participants and diabetes patients than other classes. The female patient had more bone loss compared to the male, and the posterior maxillary alveolar defect was the highest area of bone defect.

## 1. Introduction

Alveolar ridge defect refers to the loss of labial cortical or buccal cortical and medullary bone, or both, due to tooth loss, trauma during extraction, denture wear, periodontitis or congenital defects. It can be a volumetric deficit in a limited area of soft tissue and bone within the alveolar process, resulting in the soft tissue disintegrating into the bone during healing and creating contours.^[1,2]^ These contours possess difficulties in fabrication of prosthesis from an esthetic point of view.^[3]^ This is challenging for the clinician because of the esthetic demands of patients and unfavorable preexisting anatomy.^[4]^

Classifications of alveolar ridge deformities after tooth loss should aid in defining the clinical problem, predicting realistic treatment outcomes, better understanding of a particular treatment modality, and the ability to correct a clinical problem. This, in turn, could aid in proper treatment planning.^[5]^ Several classifications have been proposed; Allen et al and Seibert classified alveolar ridge deformities based on hard/soft tissue ridge defect.^[6,7]^ While Lekholm and Zarb and Misch and Judy classification was based on hard tissue ridge defect only.^[8,9]^ In 1983, Seibert classified the different types of alveolar ridge defects that a clinician may encounter while planning a prosthetic rehabilitation. It is a widely used classification of ridge defects that classifies the ridge deformation into 3 basic clinical classes: class I (horizontal bone loss), class II (vertical bone loss), and class III (both horizontal and vertical bone loss).^[7,10]^ It is a quick assessment method to evaluate the amount of alveolar ridge destruction, aid in a proper treatment plan designed for the successful prosthetic and implant restoration.^[4]^

Recently, the increasing prevalence of diabetes mellitus (DM) has made it a major chronic illness which poses a substantial threat to human health, in which patients with diabetic bone disease exhibit variable degrees of bone loss,^[11]^ slows down the rehabilitation of wounds increases the resorption of alveolar bone, and has been implicated in the unsuccessful outcome of dental treatments.^[12,13]^ So, DM patients should be motivated to practice good oral hygiene and manage their blood sugar levels appropriately to prevent bone loss.^[12]^

Cone-beam computed tomography (CBCT) in prosthetic and implant dentistry has ability to acquire detailed volumetric image data of the maxillofacial region for diagnostic and presurgical planning purposes.^[13,14]^ Successful dental implant placement for restoration of edentulous sites depends on the quality and quantity of alveolar bone available in all spatial dimensions.^[15]^

Several studies used Seibert’s classification for edentulous ridge. Deeksheetha et al; Shahroom and Jain concluded that class III, where there is both apico-coronal and buccolingual defect, was the most commonly observed type of alveolar ridge defect, followed by class I.^[16,17]^ Das et al found that class I ridge defects and normal ridges are most prevalent in all genders with slight deviation with age.^[18]^ Seibert in 1983, concluded that normal ridge had the most significant number of incidences, followed by class I and class II with least prevalence was class III.^[7,10]^ The total alveolar bone loss was greater in mandible as compared with maxilla and females had more bone loss as compared to males.^[19]^

Also, males and patients aged 40 to 49 years show a high prevalence of alveolar ridge deformities and defects.^[7,10]^ Xie et al observed that females exhibit greater alveolar ridge resorption compared with males.^[20]^ Male patients were more commonly affected than female patients, while the most common age group was 31 to 40 years of age.^[16]^

Several studies have been conducted to recognize the relation between DM and bone resorption. Wu et al found that diabetic bone disease is closely related to a decrease in bone formation and an increase in bone resorption.^[11]^ Tabassum in 2024 and Mistry et al concluded that residual ridge resorption is more in diabetic patients when compared to nondiabetic patients.^[12,21]^ DM patients have twice the amount of alveolar ridge resorption compared to controls.^[22]^ Also, Sawai et al concluded that the resorption of alveolar ridge in partially edentulous patients and patients who were subjected to dental implant placement was greater in diabetic patients.^[23]^

No previous studies have examined the alveolar ridge deformities before prosthetic and implant treatments were conducted among Saudi populations in general, and particularly in southern cities. Thus, this study was performed to assess the correlation between diabetic patients and the pattern of bone defect according to Seibert classification among patients who attended to replace their missing teeth with dental implants.

## 2. Materials and methods

### 2.1. Study design and ethical considerations

This clinical perspective CBCT study was approved by the Ethical Committee of King Khalid University, College of Dentistry under No. IRB/KKUCOD/ETH/2023-24/052 on May 30, 2024. The methods were performed in accordance with the Declaration of Helsinki.^[24]^ All participants were informed about the aims of the study. Written informed consent was obtained from all participants before their enrollment in the study. The collected information remained anonymous, and confidentiality of data was assured.

### 2.2. Inclusion and exclusion criteria

This study included both male and female participants aged 20 years and above who attended dental clinics to replace missing teeth. The inclusive criteria were cases with single or multiple partially edentulous sites, anterior or posterior ridge or bone defect, in maxillary or mandibular arches, and patients with or without a diabetic history. Patients aged <20 years old and completely edentulous were excluded from the current study.

### 2.3. Study population, sample size, and sampling technique

The present CBCT study was conducted in private dental clinics in the Aseer region, Saudi Arabia. All participants complained of missing teeth and wanted to replace them with a dental implant. The data were collected from patients’ files between May 2022 and June 2024.

To determine an appropriate sample size, we used the total number of patients needed to replace missing teeth (88,000) with a confidence level of 95% and a margin of error of 5%. Using a standard sample size calculation formula for a limited population that needs replacement of missing teeth, we calculated that a sample size of approximately 1488 files of a partially edentulous population would be representative. Therefore, a convenience sample of 1488 files of a single or multiple partially edentulous sites was targeted for the current CBCT study.^[25,26]^

### 2.4. Screening sheet and data collection process

The selected or involved files with their CBCT participants in the study were arranged and classified according to alveolar bone status based on Siebert’s classification of bone defects and the medical condition of patients in relation to diabetic mellitus. All selected data were collected from the files, including their CBCT images.

The variables recorded were patient gender “male or female”, age groups “20 to 40 years, 41 to 60 years, ≥61 years, area or site of bone defect “anterior or posterior”, arche type “maxillary or mandibular”, and Siebert’s classification of bone defects recorded as “normal, class I, class II, class III”. Patient data in relation to DM and the disease history were documented as “Yes or No” and duration of medical history with diabetes “1 to 4 years, 5 to 8 years, >8 years” were recorded.

**Figure 1.**
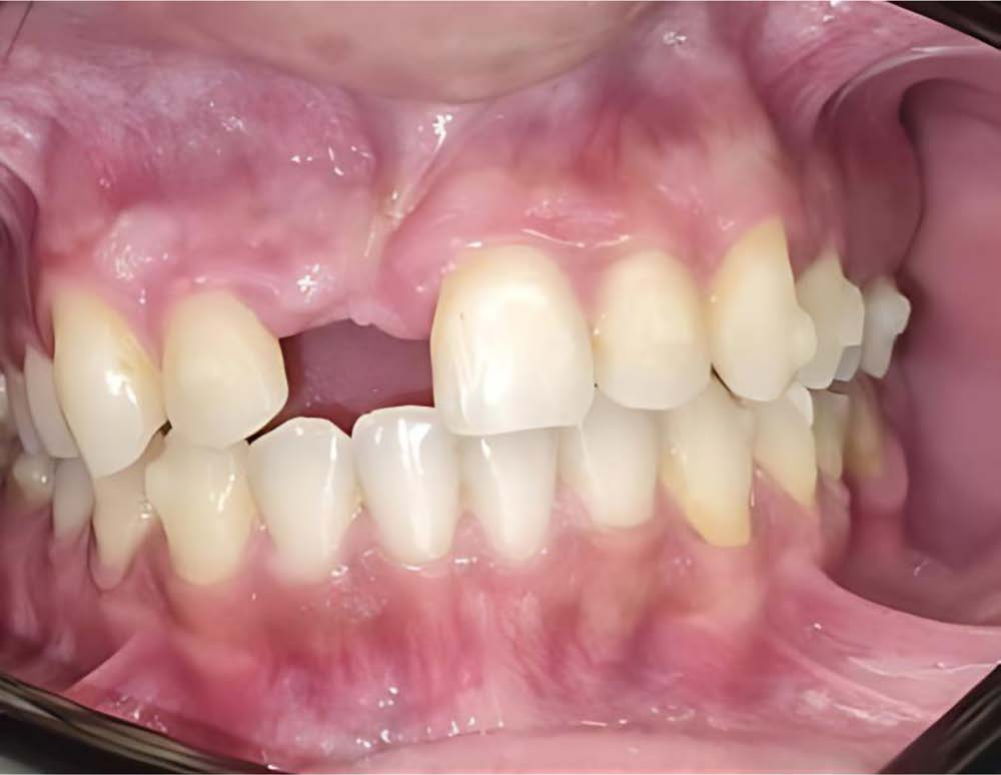
Male patient with normal bone defect at maxillary anterior.

In 1983, Seibert classified the different types of alveolar ridge defects that a clinician may encounter while planning a prosthetic rehabilitation. His classification described the following 3 clinical situations.^[7,10]^ Siebert’s classification of bone defects was classified as follows:

Normal: When an average alveolar bone loss of 1.5 to 2 mm (vertical) and 40% to 50% (horizontal) occurs within 6 months after tooth extraction^[15,27,28]^ (Fig. 1).

**Figure 2.**
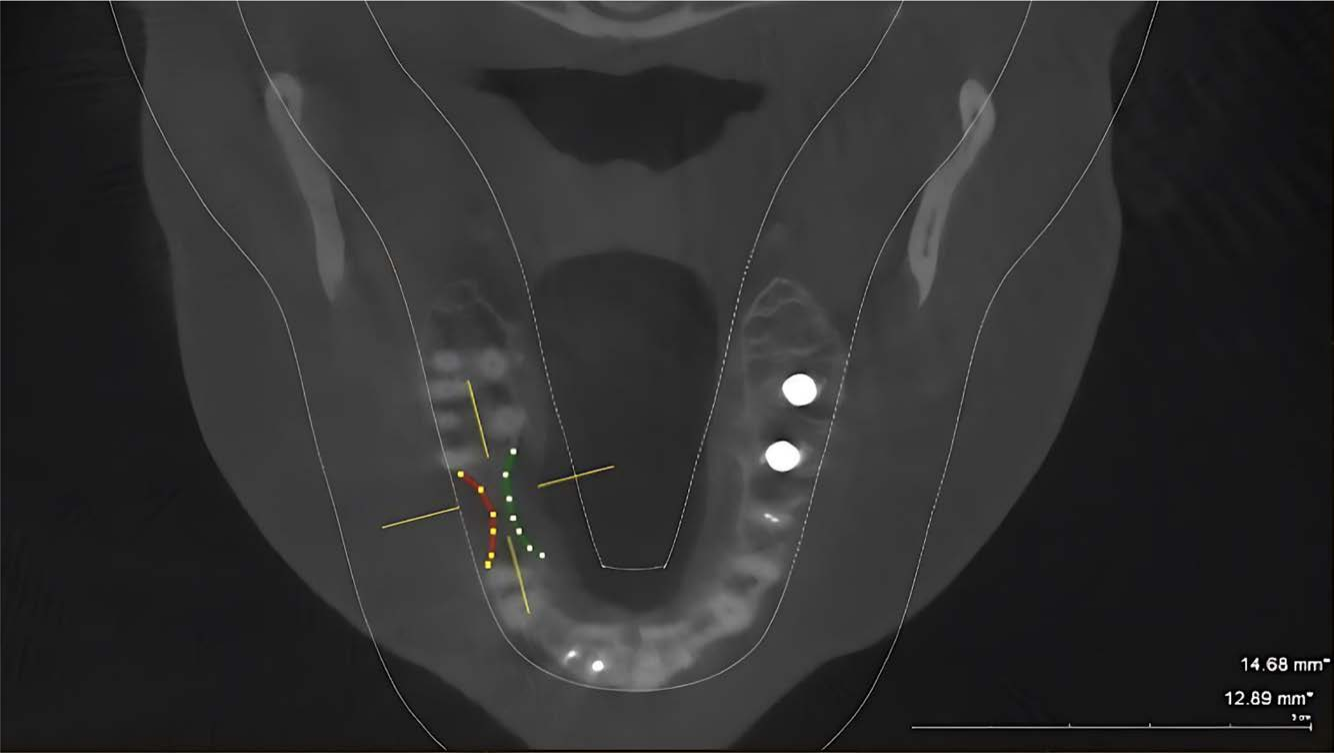
CBCT for Siebert’s classification bone defects at mandibular right class I for female patient. CBCT = cone beam computed tomography.

Class I: When ridge defects are present, there is horizontal bone loss with normal or adequate ridge height, which leads to insufficient bone volume for successful placement of regular diameter implants (buccolingual loss of tissue; Fig. 2).

**Figure 3.**
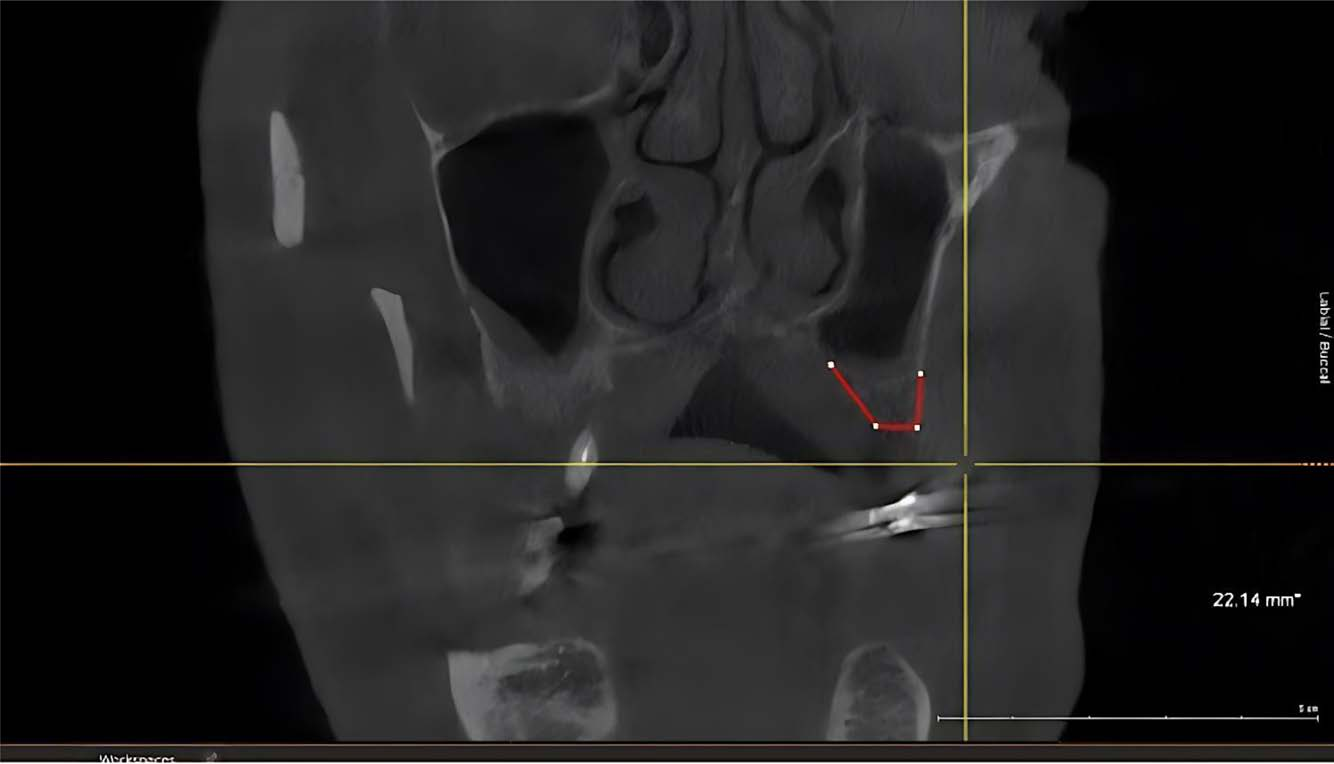
CBCT as Siebert’s classification bone defects at maxillary left class II for female patient. CBCT = cone beam computed tomography.

Class II: When there is vertical alveolar ridge bone loss with normal and adequate width, which leads to insufficient bone volume for proper positioning of regular length implants in correct prosthetic corono-apical position (apicoronal loss of tissue; Fig. 3).

**Figure 4.**
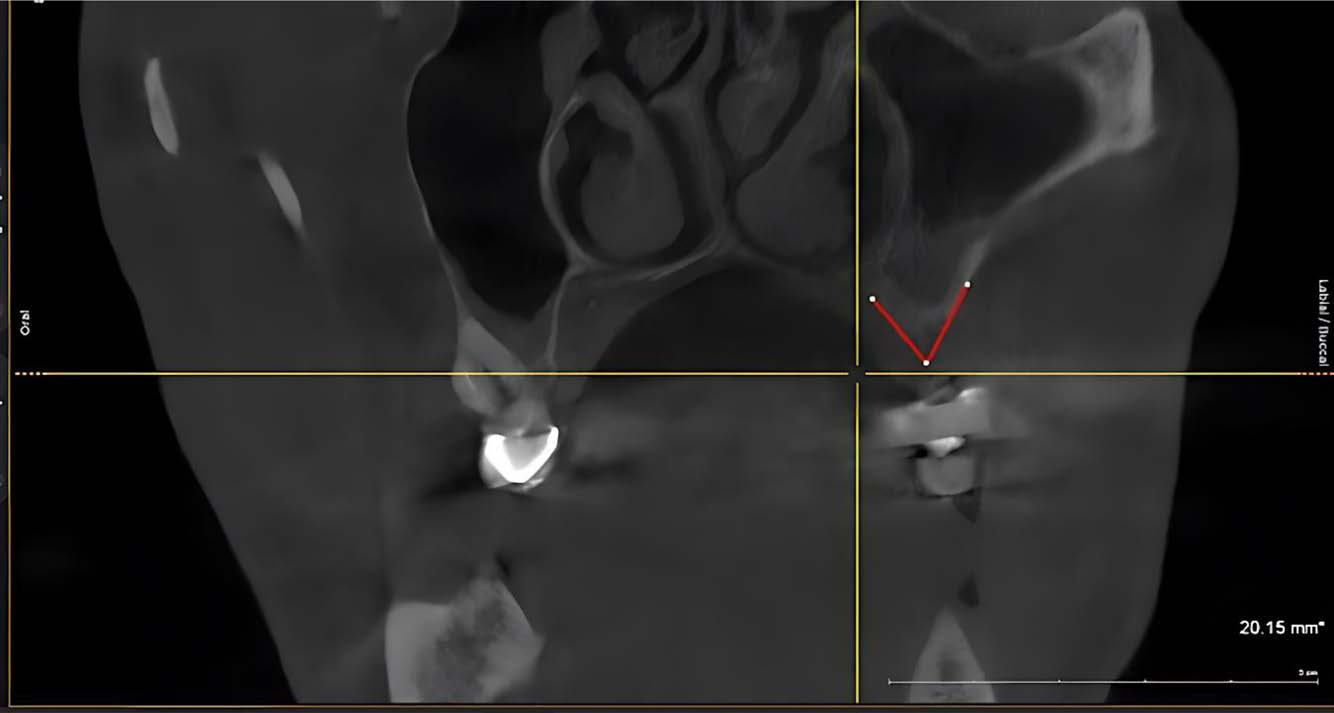
CBCT as Siebert’s classification bone defects at maxillary left class III for male patient. CBCT = cone beam computed tomography.

Class III: When there is both vertical and horizontal ridge defects with height and width of bone loss that prevents placement of successful implants in all spatial dimensions (buccolingual and apicoronal loss of tissue; Fig. 4).

### 2.5. Statistical analysis

Data were analyzed using the statistical software R version 4.2.2 (R Foundation for Statistical Computing) and Microsoft Excel. Categorical variables were represented by frequency and percentages. The association between the variables with Siebert’s classification of bone defects and in relation to the presence of DM and its duration or period of existence. Chi-square test was used to check the association between categorical variables. A *P*-value < .05 was considered statistically significant.

## 3. Results

The participants’ age ranged from 20 to 99 years old. Out of the total number of participants (1500), most of them were females 1122 (74.8%) and nondiabetic 1125 (75.0%). The highest age group was from 41 to 60 years and counted 663 (44.2%). Posterior maxillary followed by mandibular arches were the highest area of bone defect with 600 (40.0%) and 528 (35.2%), respectively. In addition, according to Siebert’s classification class II bone defect was the highest type recorded among participants, with 678 (45.2%), Table 1.

**Table 1:** Characteristics of participants (n = 1500).

Parameter	Category	Number	Percentage (%)
Diabetic patient	Yes	375	(25.0)
	No	1125	(75.0)
Gender	Male	378	(25.2)
	Female	1122	(74.8)
Age groups	20–40 yr	342	(22.8)
	41–60 yr	663	(44.2)
	>60 yr	495	(33.0)
Area of bone defect	Anterior maxillary	189	(12.6)
	Posterior maxillary	600	(40.0)
	Anterior mandibular	183	(12.2)
	Posterior mandibular	528	(35.2)
Siebert’s classification of bone defects	Normal	45	(3.0)
	Class I	369	(24.6)
	Class II	678	(45.2)
	Class III	408	(27.2)

The distribution of the 375 diabetic patients showed that 252 (67.2%) were males. Additionally, 159 (42.4%) belong to the 41 to 60 years age group. Most of them were diabetic for 5 to 8 years, making up 225 (60.0%) of the total. The most defective bone was reported in the maxillary posterior area, which made up 180 (48.0%) of the total, and class II Siebert’s classification of bone defects was the most common defect and counted as 192 (51.2%), as shown in Table 2.

**Table 2:** Patient characteristics in relation to diabetes included in this study (n = 375).

Parameter	Category	Number	Percentage (%)
Diabetic patient gender	Male	123	(32.8)
	Females	252	(67.2)
Diabetic patients with age groups	20–40 yr	78	(20.8)
	41–60 yr	159	(42.4)
	>60 yr	138	(36.8)
Number of years with diabetic	1–4 yr	30	(8.0)
	5–8 yr	225	(60.0)
	>8 yr	120	(32.0)
Area of bone defect	Anterior maxillary	60	(16.0)
	Posterior maxillary	180	(48.0)
	Anterior mandibular	24	(6.4)
	Posterior mandibular	111	(29.6)
Siebert’s classification of bone defects for diabetic patient	Normal	3	(0.8)
	Class I	21	(5.6)
	Class II	192	(51.2)
	Class III	159	(42.4)

Chi-square test was used to test the correlation between the different variables and Siebert’s classification of bone defects, as shown in Table 3. Class II bone defect was the highest among males and females, accounting for 210 (14.0%) and 468 (31.2%), respectively. Also, it was the highest in all age groups, with counts of 147 (9.8%), 330 (22.0%), and 201 (13.4%), respectively, without significant differences (*P* = .172). Class II was highest in posterior area of both arches and recorded 318 (21.2%) and 270 (18.0%), while class III was the predominant in anterior area of maxillary and mandibular arch with 69 (4.6%) and 66 (4.4%). Among diabetic patients, class II followed by class III were the highest, accounting for 192 (12.8%) and 159 (10.6%), respectively. Significant differences were recorded in variables as gender, area of bone defect, and diabetic status of the patient with *P*-values .004, .000, and .000, respectively (Table 3).

**Table 3:** Correlation between gender, age groups, area of the bone defect, diabetes status, and pattern of ridge bone defect according to Siebert’s classification, Chi-square test (n = 1500).

Parameter	Siebert’s classification of bone defects			
	Normal	Class I	Class II	Class III
Gender				
Male	0 (0.0%)	63 (4.2%)	210 (14.0%)	105 (7.0%)
Female	45 (3.0%)	306 (20.4%)	468 (31.2%)	303 (20.2%)
*P*-value	.004			
Age group				
20–40 yr	3 (0.2%)	81 (5.4%)	147 (9.8%)	111 (7.4%)
41–60 yr	21 (1.4%)	168 (11.2%)	330 (22.0%)	144 (9.6%)
>60 yr	21 (1.4%)	120 (8.0%)	201 (13.4%)	153 (10.2%)
*P*-value	.172			
Area of bone defect				
Anterior maxillary	6 (0.4%)	66 (4.4%)	48 (3.2%)	69 (4.6%)
Posterior maxillary	6 (0.4%)	102 (6.8%)	318 (21.2%)	174 (11.6%)
Anterior mandibular	15 (1.0%)	60 (4.0%)	42 (2.8%)	66 (4.4%)
Posterior mandibular	18 (1.2%)	141 (9.4%)	270 (18.0%)	99 (6.6%)
*P*-value	.000			
Diabetic status				
Yes 375 (25%)	3 (0.2%)	21 (1.4%)	192 (12.8%)	159 (10.6%)
No 1125 (75%)	42 (2.8%)	348 (23.2%)	486 (32.4%)	249 (16.6%)
*P*-value	.000			

The association between the diabetic status of the participants and Siebert’s classification of bone defects is presented in Table 4. Class II and III were the more predominant in males, with 60 (16.0%), while class II was prevalent among females, 132 (35.2%). The same types of Siebert’s classification of bone defects were accounted as common among different age groups, with class II being higher among the middle-aged group and class III being higher in the elderly group. Class II followed by class III were more common in the posterior area and counted 102 (27.2%) and 69 (18.4%) for maxillary arch, while it was counted as 63 (16.8%) and 39 (10.4%) for mandibular arch. Chi-square test shows no significant differences for variables as gender, age groups, and area of bone defect with *P*-values of .611, .708, and .191, respectively. Regarding the duration of the participants and diabetes, class II was the highest with 5 to 8 years and counted as 159 (42.4%), and class III was the highest and associated with higher numbers of diabetes patients and recorded as 90 (24.0%) with significant differences (*P* = .000), Table 4.

**Table 4:** Association of diabetes patients with gender, age groups, area of the bone defect, number of years with diabetes, and pattern of bone ridge defect according to Siebert’s classification, Chi-square test (n = 375).

Parameter	Siebert’s classification of bone defects			
	Normal	Class I	Class II	Class III
Gender				
Male	0 (0.0%)	3 (0.8%)	60 (16.0%)	60 (16.0%)
Female	3 (0.8%)	18 (4.8%)	132 (35.2%)	99 (26.4)
*P*-value	.611			
Age group				
20–40 yr	0 (0.0%)	6 (1.6%)	36 (9.6%)	36 (9.6%)
41–60 yr	3 (0.8%)	9 (2.4%)	93 (24.8%)	54 (14.4)
>60 yr	0 (0.0%)	6 (1.6%)	63 (16.8%)	69 (18.4%)
*P*-value	.708			
Area of bone defect				
Anterior maxillary	3 (0.8%)	3 (0.8%)	21 (5.6%)	33 (8.8%)
Posterior maxillary	0 (0.0%)	9 (2.4%)	102 (27.2%)	69 (18.4%)
Anterior mandibular	0 (0.0%)	0 (0.0%)	6 (1.6%)	18 (4.8%)
Posterior mandibular	0 (0.0%)	9 (2.4%)	63 (16.8%)	39 (10.4%)
*P*-value	.191			
Number of years with diabetes				
1–4 yr	3 (0.8%)	15 (4.0 %)	6 (1.6%)	6 (1.6%)
5–8 yr	0 (0.0%)	3 (0.8%)	159 (42.4%)	63 (16.8%)
>8 yr	0 (0.0%)	3 (0.8%)	27 (7.2%)	90 (24.0%)
*P*-value	.000			

## 4. Discussion

A localized residual alveolar ridge defect is characterized by deficiency of bone volume and soft tissue collapse during healing creating unesthetic contours.^[29,30]^ Besides, it may also lead to food impaction and difficulty in speech due to percolation of saliva.^[31]^ It is important to close the ridge defect by replacing the tooth loss and to achieve good esthetics, phonetics and mastication.^[32]^ So, this study was conducted to assess correlation between diabetic patients and pattern of bone defect according to Seibert classification among patients who attended to replace their missing teeth with dental implants.

In this study, class II bone defect according to Siebert’s classification was the highest type recorded among participants (45.2%), followed by class III (27.2%). This is in contrast to the American study by Seibert and previous findings among Indian populations by Deeksheetha et al, Shahroom and Jain, Das et al, and other studies.^[7,10,16,17,32]^ While the largest age group was from 41 to 60 years with (44.2%), the posterior maxillary was the area recorded with the highest number of bone defects (40.0%). Those findings differed from percentages reported earlier among the Indian population by Deeksheetha et al, Das et al, and Acharya et al.^[16,18,19]^

Most of the diabetic patients were females (67.2%), this result is in parallel with previous findings among the Jordan population by Al-Jabrah^,[25]^ but in contrast with findings among Saudi diabetic patients by Tabassum^.[12]^ The most affected bone was the maxillary posterior area (48.0%). Among diabetic patients, class II defects were the highest with 51.2%, then class III defects with 42.4%. Significant differences were recorded in variables as gender, area of bone defect, and diabetic status of the patient.

Amongst all the classifications for the alveolar ridge defects, Seibert’s classification was chosen for the present study as it is the most commonly used due to its ease of classification in clinical practice.^[16]^ Siebert’s classification is a quick assessment method to evaluate the amount of alveolar ridge destruction so that a proper treatment plan can be designed for the successful prosthetic restoration.^[4]^

In the current study, class II bone defect according to Siebert’s classification was the highest type recorded among participants with (45.2%) followed by class III represent (27.2%), this result is in contrast to Deeksheetha et al, who found that the least commonly observed type of alveolar ridge defect was class II, while class III was the most commonly observed type of alveolar ridge defect followed by class I.^[16]^ Different results were found by Das et al, who found that class I ridge defects and normal ridges are most prevalent in all 3 genders (male, female and transgender).^[18]^ Similarly, Seibert also found that normal ridges had the greatest number of incidences among different ridge defect classes.^[7,10]^

According to the findings from the collected data, the posterior maxillary alveolar defect was the highest area of bone defect, with 40.0%. Acharya et al, findings differed from our results they found that the total alveolar bone loss was greater in mandible as compared to maxilla. However, their observation matched our result that females had more bone loss as compared to males, and alveolar bone loss was seen more in the posterior region as compared to the anterior region because of masticatory forces.^[19]^ The mandible showed significant bone loss (*P* < .05) as compared to the maxilla. There was significantly (*P* < .05) more bone loss in females as compared with males.

From the results of this study, the highest age group with defects was from 41 to 60 years and counted (44.2%). Deeksheetha et al, results differed from our findings; they found the most affected age group was the 31 to 40 years of age with (29.24%) and male patients were more commonly affected than female patients.^[16]^ However, there is no significant difference between Seibert’s classification and gender. But Seibert found a different result compared to the present study, that male gender and patients aged 40 to 49 years show a high prevalence of alveolar ridge deformities and defects.^[7,10]^ Similarly, a study by Das et al, found that male gender had a high prevalence of alveolar ridge deformities, with 54.5% and patients aged 40 to 49 years old had a high incidence of alveolar ridge defects, with 50.9%.^[18]^ Also, the majority of the male participants had class I ridge pattern compared to female participants.^[18]^ Similar significant differences were documented in the present study.

It has been discovered that DM slows down wound healing, increases the resorption of alveolar bone, and has been implicated in the compromised outcome of replacing teeth.^[33,34]^ DM is a disease resulting from impaired insulin availability in the body, leading to increased blood glucose which is called as (type 1 DM), or varying degree of insulin resistance or unable to use the available insulin by the body, which is called (type 2 DM). Uncontrolled diabetes is one of the main reasons for accelerated residual ridge resorption.^[32]^ The studies suggested that patients with uncontrolled or poorly controlled type 2 DM had a higher chance of residual ridge resorption and more severe bone loss when compared to individuals who didn’t have diabetes.^[35]^ The patients with type 2 DM were positively associated with an increased risk of a change in bone score compared to subjects without diabetes.^[36]^

Wu et al, in 2022, found that diabetic bone disease is closely related to a decrease in bone formation and an increase in bone resorption.^[11]^ Also, the same result was found by Tabassum in 2024 and Mistry et al in 2020, they concluded that residual ridge resorption was twice as much in diabetic patients compared to nondiabetic patients. The results showed that females had significantly more resorption than males, with statistically significant differences (*P* < .001).^[12,21]^ Also, statistically significant gender differences in mandibular ridge resorption have been recorded in the DM (*P* < .001) and control (*P* < .05) groups.^[22]^ Similar to the results of the present study, a study by Sawai et al, in 2024, concluded that the resorption of the alveolar ridge in partially edentulous patients and patients who were subjected to dental implant placement was greater in diabetic patients.^[23]^

No previous studies examined the correlation between DM and bone defect according to Seibert classification; however, this was examined in the present study. In the present study, we found that among diabetic patients, class II defects were the most common at 51.2%, followed by class III at 42.4%. Diabetic females with class II ridge defects accounted for 31.2%, which was higher than that of males at 14%. The highest age group of diabetic patients with Class II defects was 41 to 60 years old at 22%, and the most affected area of bone defect was the posterior maxilla at 21.2%. Significant differences were recorded in variables as gender, area of bone defect, and diabetic status of the patient with *P*-values .004, .000, and .000, respectively. A noteworthy influence of age and gender on the risks of alveolar bone loss when combined with diabetes conditions.^[37]^

The main limitation of the current study was a limited sample size and geographic restriction. In the future, further studies can be done on a large population incorporating the data from different universities within the state and country, as a multi-center study. Seibert’s classification represents the 3 broad categories of ridge defects, but a division of those defects into subcategories is lacking. Each of the 3 classifications can be further subdivided into categories based on the size of the defect. Such division may prove useful in selecting treatment modalities and predicting treatment outcomes.

## 5. Conclusion

From this CBCT radiographic study, we can conclude that according to Seibert’s classification, class II followed by class III bone defect was the highest type recorded among all participants and diabetic participants. The female patient had more bone loss as compared to the male, and the posterior maxillary alveolar defect was the highest area of bone defect in both participants and diabetic patients. Significant differences were recorded in variables as gender, area of bone defect, and diabetic status of the patient.

## Author contributions

**Conceptualization:** Manea Musa M. Alahmari.

**Data curation:** Manea Musa M. Alahmari.

**Formal analysis:** Manea Musa M. Alahmari.

**Funding acquisition:** Manea Musa M. Alahmari.

**Investigation:** Manea Musa M. Alahmari.

**Methodology:** Manea Musa M. Alahmari.

**Project administration:** Manea Musa M. Alahmari.

**Supervision:** Manea Musa M. Alahmari.

**Validation:** Manea Musa M. Alahmari.

**Visualization:** Manea Musa M. Alahmari.

**Writing – original draft:** Manea Musa M. Alahmari.

**Writing – review & editing:** Manea Musa M. Alahmari.
